# Does timing matter? Unlocking the value of repeated MRI in the early phases after severe traumatic brain injury

**DOI:** 10.1007/s00701-026-06962-9

**Published:** 2026-06-20

**Authors:** Daniel Pinggera, Philipp Geiger, Christian Preuss-Hernandez, Ronny Beer, Claudius Thomé, Ondra Petr

**Affiliations:** 1 Department of Neurosurgery, Medical University Innsbruck, Anichstraße 35, 6020 Innsbruck, Austria; 2 Department of Neurology, Medical University Innsbruck, 6020 Innsbruck, Austria

**Keywords:** Brain injuries, Acute traumatic subdural hematoma, Magnetic resonance imaging, Prognosis

## Abstract

**Background:**

Cerebral MRI after severe traumatic brain injury (sTBI) may refine lesion characterization and support prognosis, but the optimal timing within the early course remains uncertain.

**Methods:**

We retrospectively analyzed 17 adult sTBI patients (GCS ≤ 8) who underwent two clinically indicated 3 T MRI examinations: an early scan (≤ 72 h) and a subacute scan (day 12–14). Three board-certified neurointensivists, blinded to scan timing, independently rated lesion burden across standard sequences (T1, T2/FLAIR, SWI, DWI) and evaluated (i) comparative clinical utility of early vs subacute MRI, (ii) imaging-only prognosis (expected favorable vs unfavorable functional outcome, dichotomized by Glasgow Outcome Scale), and (iii) whether imaging findings would be expected to prompt a management change. Inter-rater agreement was quantified by ICC (lesion counts) and Fleiss’ kappa (categorical ratings). The patient (MRI-pair) was the statistical unit; rater-level ratings were used for reliability estimation, not as independent observations.

**Results:**

At the patient level, early and subacute MRI were judged equally useful in 12/17 (70.6%) cases (Fleiss’ kappa for pairwise utility rating: 0.33). Lesion burden demonstrated substantial overlap between time points; paired comparisons of rater-averaged lesion counts did not indicate a systematic difference between early and subacute MRI across T1, T2/FLAIR, SWI, or DWI (all *p* > 0.05). Monitoring-related artifacts were frequent but rarely reduced overall interpretability.

**Conclusions:**

In this selected cohort able to undergo two MRIs within the early course of sTBI, early and subacute MRI provided largely concordant information. MRI timing should be individualized based on clinical stability and the specific diagnostic question; routine repetition within the first two weeks may not be necessary.

## Introduction

Severe traumatic brain injury (sTBI) remains a leading cause of mortality and long-term disability globally, with significant impacts on quality of life and economic burden. Magnetic resonance imaging (MRI) can play an important role in sTBI management, offering superior sensitivity in detecting various traumatic lesions compared to conventional imaging modalities. Recent evidence suggests that MRI can identify abnormalities in approximately 30% of cases where computed tomography (CT) appears normal, particularly in detecting subtle contusions, microhemorrhages, and axonal injuries [1, 4].

However, performing early MRI in sTBI patients presents significant challenges, including safety concerns related to neuromonitoring equipment and risks associated with intrahospital transport of critically ill patients. The requirement for neuromonitoring devices, essential for tracking intracranial pressure (ICP) and brain tissue oxygenation (PbtO2), introduces technical complications due to potential probe-related heating in high-magnetic-field MRI environments [7, 17]. Furthermore, the transportation of ventilated sTBI patients for imaging presents substantial risks. Clinical studies have documented adverse events during intrahospital transport, including hemodynamic instability and potential equipment-related complications [6, 12, 13].

The ideal timing of MRI following sTBI remains a subject of debate. Early MRI (≤ 72 h post-injury) may capture primary injury-related structural damage, while subacute MRI (12–14 days post-injury) could reveal secondary injury processes. This diagnostic advantage must be balanced against the significant practical challenges and risks involved. Too early diagnostic imaging despite clinical instability may pose a risk for long-term neurological outcome via substantial blood-pressure and ICP crises with subsequently reduced cerebral perfusion. MRI imaging with extensive sequence-protocols may take up to one hour duration – capability of the patient to tolerate this duration should be ensured first [3, 16].

This study aims to evaluate the comparative diagnostic utility of MRI at two critical time points: within 72 h and between days 12–14 post-injury. We hypothesize that this temporal comparison will elucidate whether significant differences exist between the two imaging time frames and if one should be preferred over the other for clinical decision making, using a paired within-patient design.

## Material and methods

This investigation comprises a retrospective analysis of prospectively collected imaging data in sTBI patients, incorporating advanced MRI protocols at two distinct time points to evaluate traumatic lesion evolution. Patients aged 18 to 85 years with sTBI (Glasgow Coma Scale score ≤ 8) requiring management per Brain Trauma Foundation guidelines, including multimodal neuromonitoring, were included. Seventeen patients met the inclusion criteria of having severe TBI, being older than 18 years and being in a sufficient clinical state to lie supine for an hour without threatening ICP and blood pressure crises. Patients were excluded based on standard MRI contraindications or clinical instability leading to abortion of the MRI examination. The protocol received ethical approval (AN 1443/2021) with informed consent obtained from participants or legal representatives (AN2014-0201 339/4.6). Clinical imaging was obtained as institutional routine dictated, but was analyzed for research purposes. 31P-MR-Spectroscopy (MRS) was additionally obtained.

### Study population and patient selection

This analysis focuses on patients with paired MRI examinations within two prespecified early windows after sTBI. MRI in our institution is not performed routinely in every sTBI patient but is considered after interdisciplinary discussion when (a) incremental diagnostic/prognostic value beyond CT is expected and (b) transport and prolonged scanning are deemed safe (hemodynamic stability, controllable ICP, MRI compatibility of monitoring equipment).

Inclusion criteria were: age 18–85 years; sTBI (GCS ≤ 8); management according to Brain Trauma Foundation guideline-based neurocritical care including multimodal neuromonitoring; completion of two MRI examinations within the defined windows (≤ 72 h and day 12–14) with complete standard sequences (T1, T2/FLAIR, SWI, DWI).

Exclusion criteria were: standard MRI contraindications; clinical instability precluding safe transport or completion of the scan; aborted MRI; MRI performed outside the prespecified time windows; and any major neurosurgical intervention between the two scans that would confound temporal lesion comparison.

Patients who underwent only one MRI within the early course (early-only or subacute-only) or no MRI were not eligible for this paired comparison. The main reasons for missing one time point were clinical instability, death or transfer before follow-up imaging, or MRI contraindications. This selection process is explicitly considered in the limitations as it enriches for clinically stable sTBI survivors and may reduce generalizability.

### MRI protocol and sequences

Imaging was conducted on a 3-Tesla whole-body system (Verio, Siemens Medical AG) utilizing:

- 3D T1 MPRAGE
- 3D T2-space/FLAIR
- Diffusion-weighted imaging
- 3D susceptibility-weighted imaging

MRI acquisition was performed at two predetermined intervals: the early phase, within 72 h post-injury, and the subacute phase, 12–14 days post-trauma. Radiology reports of the included patients were checked for substantial deviations of interpretation. The MRI acquisition protocol was identical at both time points and corresponded to the protocol previously published in our 31P-MR-spectroscopy study in severe TBI [14], where detailed acquisition parameters are reported.

### Rater assessment and operational definitions

Three board-certified neurointensivists independently reviewed all MRI examinations. Raters were blinded to clinical data and to the acquisition time point; scans were presented in randomized order. The aim of the rating was to capture the *neurocritical care* interpretation of MRI findings beyond formal neuroradiology reporting. Accordingly, board-certified neurointensivists were intentionally selected as raters to reflect real-world neurocritical care decision-making.

Lesion burden was assessed per sequence (T1, T2/FLAIR, SWI, DWI) by counting discrete traumatic lesions (e.g., contusions/hemorrhagic lesions, microbleeds on SWI, diffusion-restricted lesions consistent with traumatic ischemia/axonal injury) as separate foci; lesions extending over multiple adjacent slices were counted once. Across the cohort, relevant lesion categories included epidural hematoma, acute and chronic subdural hematoma, intracerebral hemorrhage/contusions, diffuse axonal injury, traumatic subarachnoid hemorrhage, intraventricular hemorrhage, and fractures. The following categorical outcomes were operationalized as follows:

1. Comparative clinical utility of MRI at both time points (pairwise utility): After reviewing both scans of the same patient (still blinded to timing), raters judged whether the *overall actionable information content* was equivalent between the two scans. “Utility” was defined as the ability of the MRI to answer neurocritical care–relevant questions beyond CT and bedside monitoring, specifically: detection of occult lesions (e.g., diffuse axonal injury/brainstem involvement, microhemorrhages, ischemia), refinement of injury extent, and contribution to prognosis- and goal-of-care discussions. Ratings were recorded as equally useful vs not equally useful (i.e., one scan provided clinically relevant additional information compared with the other).
2. Imaging-only prognosis classification: For each MRI, raters classified the expected long-term functional outcome based solely on MRI patterns known to be associated with outcome after moderate-to-severe TBI (e.g., extent/location of diffuse axonal injury, brainstem involvement, large bilateral lesions, extensive diffusion restriction/ischemia). Prognosis was dichotomized as favorable vs unfavorable in alignment with the Glasgow Outcome Scale framework (favorable: GOS 4–5; unfavorable: GOS 1–3). This rating reflects an imaging-based expectation and does not replace multimodal clinical prognostication.
3. Likelihood of treatment adjustment based on imaging findings: For each MRI, raters answered whether the findings would be expected to trigger a change in ICU management compared with what would be done based on CT and standard monitoring alone (yes/no). “Treatment adjustment” was defined pragmatically and could include escalation/de-escalation of ICP/CPP targets, additional surgical consultation, targeted follow-up imaging, changes in antithrombotic management, seizure management, or earlier rehabilitation/goal-of-care discussions. This item was intentionally hypothetical and aimed to quantify perceived actionability of MRI findings.

### Statistics

Statistical analyses were performed using SPSS (IBM, Armonk, NY, USA); significance was set at p < 0.05. The patient (MRI-pair) was the primary unit of analysis. Ratings from three observers were used to estimate inter-rater reliability and to derive patient-level summaries; they were not treated as independent observations for between-timepoint inference.

For lesion burden, each rater provided lesion counts per sequence for each MRI. For each patient and sequence, we summarized lesion burden at each time point by averaging counts across raters, and compared early vs subacute MRI using paired non-parametric testing (Wilcoxon signed-rank) given the small sample size and count nature of the data. To visualize within-patient change, we additionally report the lesion ratio (subacute/early) per sequence.

Inter-rater reliability for lesion counts was quantified using the intraclass correlation coefficient (ICC) with a two-way random-effects model and absolute agreement. Categorical items were summarized as proportions and inter-rater agreement was.

The three independent raters’ ratings were analyzed in two stages, depending on the scaling and nature of the data: Intraclass Correlation Coefficient was used for the radiological comparisons using a two-way random/mixed effects model. The interpretation of ICC values followed Koo and Li's guidelines [9]. For qualitative comparison the inter-rater reliability was assessed using Fleiss' Kappa, the interpretation based on Landis and Koch [11].

## Results

### Demographics and baseline characteristics

A total of 17 patients (aged 24–74 years; mean age 44.4 years; 3 females and 14 males) with sTBI were re-evaluated. The median Glasgow Coma Scale (GCS) score upon admission was 6 (range 3–8). The decision to conduct the initial MRI was made through an interdisciplinary discussion assessing the necessity and potential benefits of the diagnostic procedure according to the initial study protocol to be found elsewhere [14]. Between the two MRI scans, none of the patients underwent any surgical intervention or removal of neuromonitoring devices.

### Radiological comparison

**Fig. 1:**
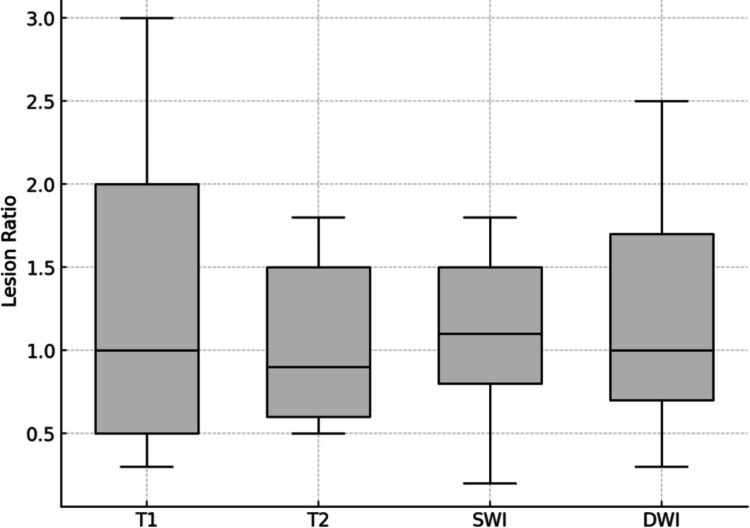
Showing the overall lesion ratio for radiological findings as assessed by all three raters

The calculated overall lesion ratio (number of lesions in the subacute phase/number of lesions in the acute phase) indicate a slight tendency toward an increase in the number of lesions (see Fig. 1). Inter-rater agreement for lesion counts was moderate-to-excellent across sequences (Table 1). Visual inspection of lesion ratios suggested no systematic shift in lesion burden between early and subacute imaging across sequences at the cohort level, while individual patients showed heterogeneous trajectories (Fig. 1). The Intraclass Correlation Coefficient (ICC) for T1, T2, SWI and DWI sequences showed a good to excellent reliability between all three observers (see Table 1).

**Table 1:** Inter-rater reliability of lesion load in MRI sequences

MRI Sequence	Mean ICC (95%-CI)	Interpretation
T1	0.89(0.75–0.96)	Excellent reliability
T2	0.63 (0.16–0.85)	Moderate reliability
SWI	0.80 (0.55–0.92)	Good reliability
DWI	0.68 (0.28–0.88)	Moderate reliability

### Relevance and usefulness of MRI findings

**Fig. 2:**
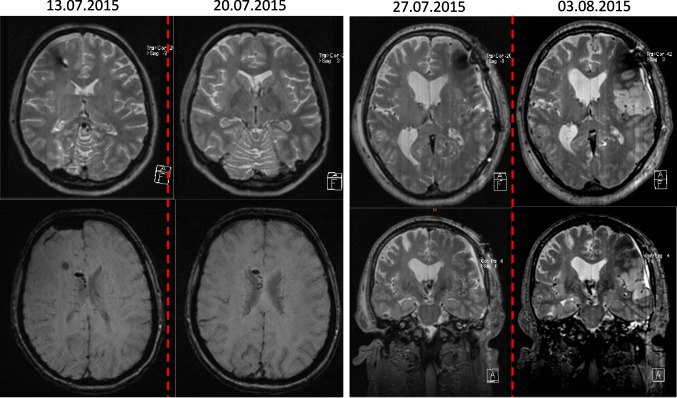
On the left: acute and subacute MRI showing little to no difference in lesion load. On the right: acute and subacute MRI showing clear dynamic on lesion load with hypointensity in the left frontotemporal region

The relevance and utility of MRI findings for clinical decision-making were evaluated using Fleiss' Kappa for categorical ratings, with results as follows (Tables 2 and 3). Examples of used MRIs are depicted in Fig. 2. To improve transparency of the underlying imaging data, we provide representative paired examples in Fig. 2. The pairwise item ‘equal helpfulness’ was evaluated at the patient level by directly comparing both scans within each MRI pair. In contrast, prognosis estimation, treatment adjustment, and monitoring-related items were rated separately for each individual MRI examination.

**Table 2:** Assessment of MRI Utility and relevance of findings by three independent raters

	Rater 1 (%)	Rater 2 (%)	Rater 3 (%)
Helpfulness of MRI for clinical decisions (% for “equal helpful”)	91	70	94
Prognosis estimation (% for “poor prognosis”)	41	47	41
Treatment adjustment post-MRI (% for “yes”)	3	15	30
Artefacts due to intracranial monitoring (% for “yes”)	12	50	12
Defects from intracranial monitoring (% for “yes”)	82	30	82
Conclusiveness affected by monitoring (% for “no”)	74	94	91

**Table 3:** Agreement across raters and Fleiss' Kappa statistics, including standard error and agreement levels

	Kappa Value	Standard Error (SE)	Agreement Level
Helpfulness of MRI for clinical decisions (% for “equal helpful”)	0.33	0.77	Fair agreement
Prognosis estimation (% for “poor prognosis”)	0.58	0.91	Moderate agreement
Treatment adjustment post-MRI (% for “yes”)	0.11	0.99	Slight agreement
Artefacts due to intracranial monitoring (% for “yes”)	0.21	0.1	Fair agreement
Defects from intracranial monitoring (% for “yes”)	0.50	0.1	Moderate agreement
Conclusiveness affected by monitoring (% for “no”)	0.77	0.1	Substantial agreement

Percentages for prognosis estimation, treatment adjustment, and monitoring-related artifacts/defects refer to scan-level ratings across 34 MRI examinations. The ‘equal helpfulness’ item reflects a patient-level pairwise comparison between the two MRI examinations of each patient.

## Discussion

Determining the ideal timing for MRI in sTBI involves balancing diagnostic utility, clinical management, and patient safety. This paired comparison suggests that, in clinically stable sTBI patients able to undergo repeated MRI within the early course, neither time point consistently outperformed the other in perceived clinical utility. Importantly, this does not imply that timing is irrelevant in unselected sTBI populations; rather, feasibility and selection (stability, survivorship) strongly shape which patients can be scanned early and/or repeatedly.

Although MRI is known to play an important role in management of severe TBI protocols and timing vary widely. To the best of our knowledge, this is among the first studies to directly compare two early MRI time points within the same severe TBI patients, thereby extending prior work from our group and complementing the broader literature on MRI timing in TBI. Our findings show comparable lesion loads across early (≤ 72 h) and subacute (12–14 days) MRI scans in T1, T2, SWI, and DWI sequences. The agreement in perceived equality of helpfulness between these time points challenges the current concept that multiple early MRI scans could be necessary for sTBI management as imaging at different phases captures varying pathophysiological processes: early MRI primarily detects structural damage from the initial injury, while subacute MRI reveals secondary processes such as edema and neuroinflammation. This dual-phase utility aligns with previous findings, such as those by Haghbayan et al., who highlighted the prognostic value of lesion patterns in moderate to severe TBI [5]. Our data revealed similar results for prognosis estimation based solely on imaging among the three raters. These findings suggest that both time points are comparably useful in this regard.

Overall, there is limited data on longitudinal comparison of MRI at two early time points. A study by Richter et al., using the CENTER-TBI database, found that ultra-early MRI (within 72 h post-injury) had superior prognostic value in mild TBI compared to later scans after two to three weeks, highlighting its ability to detect critical pathophysiological changes or structural damage affecting long-term outcomes. However, the role of MRI in moderate and severe TBI remains unclear, as mild TBI patients can typically undergo imaging earlier. Notably, a repeat MRI three weeks later showed increased ventricular volume and decreased brain volume, potentially aiding in predicting future sequelae [15].

Similar results were shown in sport-related concussion, where white matter changes in DWI were more likely to be present after 8 days than in the first 24 hours [10].

Performing MRI in critically ill sTBI patients presents significant technical challenges, particularly concerning the compatibility of neuromonitoring equipment. Risks such as probe-related heating and tissue damage in 3 T MRI environments are important safety considerations, given the essential role of neuromonitoring in managing intracranial pressure and optimizing cerebral perfusion. Despite these challenges, it was demonstrated that early MRI is feasible and can be safely integrated with neuromonitoring devices without compromising patient safety or image quality [13]. Our data indicates that, despite some artifacts and damage caused by the probes, the overall readability remains high, with strong inter-rater agreement. Also, these factors do not appear to significantly impact the prognosis or perceived helpfulness of the MRI evaluations.

From a methodological standpoint, the retrospective analysis of prospectively collected data offers a solid foundation for evaluation. The blinding of raters to MRI timing minimizes potential bias in lesion assessment and prognostic evaluation, thereby enhancing the reliability of the results. This study's robust design, involving three board-certified neurointensivists for lesion assessment, enhances the reliability of our findings. The interobserver agreement observed in our study is comparable to or exceeds that reported in similar investigations regarding neuroradiological findings [2, 8, 18]. Intraobserver reliability was not assessed in the protocol of this study. A slight variability, however, seems logical, considering the complexity of the pathologies and, particularly regarding potential treatment changes, the differing personal experiences of the evaluators. Although the paired cohort size is limited in absolute terms, it is comparatively substantial for serial MRI studies in severe TBI requiring two early examinations in the same patient. The study population, defined by strict inclusion criteria (GCS ≤ 8), represents a focused cohort of patients with severe TBI. This specificity strengthens the internal validity of the results but warrants caution when applying these findings to moderate TBI cases or pediatric populations. Future research with larger and more diverse cohorts could yield additional insights and improve the external validity of these conclusions.

This study is limited by its small sample size and selection bias: only patients stable enough to tolerate transport and prolonged MRI at two early time points were included. Patients with early-only imaging, subacute-only imaging, or no MRI (e.g., due to instability, early death, or contraindications) were not represented. Therefore, generalizability to the full spectrum of sTBI—particularly the most unstable patients—is limited. The categorical ‘treatment adjustment’ outcome was intentionally hypothetical, reflects perceived rather than actual management change, and may vary with institutional practice. In addition, lesion burden was assessed using a pragmatic counting approach intended to reflect clinical image interpretation rather than a formal neuroradiological lesion-mapping system. Sex was recorded descriptively but was not analyzed as an independent factor, as the study was not designed or powered for sex-based comparisons and the sex distribution was markedly unbalanced.

## Conclusions

In this selected cohort of sTBI patients who completed MRI both within 72 h and again at day 12–14, early and subacute MRI provided largely overlapping information, and most MRI pairs were judged similarly useful by neurointensivist raters. These findings support an individualized approach to MRI timing that prioritizes patient stability and the specific clinical question. Routine repetition of MRI within the first two weeks should be considered selectively, particularly when new clinical questions arise or when the initial study was limited by artifacts or incomplete characterization.

## Data Availability

Source data is obtainable by contacting the corresponding author.

## Declarations

### Animal studies

Not applicable – no animal studies were conducted.

## References

[1] Capizzi A, Woo J, Verduzco-Gutierrez M (2020) <article-title update="added">Traumatic brain injury. Med Clin North Am 104(2):213–238. 10.1016/j.mcna.2019.11.001 32035565 10.1016/j.mcna.2019.11.001

[2] Geerdink N, van der Vliet T, Rotteveel JJ, Feuth T, Roeleveld N, Mullaart RA (2012) Essential features of Chiari II malformation in MR imaging: an interobserver reliability study- -part 1. Childs Nerv Syst 28(7):977–85. 10.1007/s00381-012-1761-5 22547226 10.1007/s00381-012-1761-5 PMC3376258

[3] Geiger P, Gmeiner R, Schon V, Petr O, Thome C, Pinggera D (2025) Timing of magnetic resonance imaging (MRI) in moderate and severe TBI: a systematic review. J Clin Med. 10.3390/jcm14124078 40565823 10.3390/jcm14124078 PMC12194093

[4] Haghbayan H, Boutin A, Laflamme M et al (2017) The Prognostic Value of MRI in Moderate and Severe Traumatic Brain Injury: A Systematic Review and Meta-Analysis. Crit Care Med 45(12):e1280–e1288. 10.1097/CCM.0000000000002731 29028764 10.1097/CCM.0000000000002731

[5] Haghbayan H, Boutin A, Laflamme M et al (2017) The prognostic value of MRI in moderate and severe traumatic brain injury: a systematic review and meta-analysis. Crit Care Med. 10.1097/CCM.0000000000002731 10.1097/CCM.0000000000002731 29028764

[6] Hawryluk GWJ, Citerio G, Hutchinson P et al (2022) Intracranial pressure: current perspectives on physiology and monitoring. Intensive Care Med 48(10):1471–1481. 10.1007/s00134-022-06786-y 35816237 10.1007/s00134-022-06786-y

[7] Kawoos U, McCarron RM, Auker CR, Chavko M (2015) Advances in intracranial pressure monitoring and its significance in managing traumatic brain injury. Int J Mol Sci 16(12):28979–28997. 10.3390/ijms161226146 26690122 10.3390/ijms161226146 PMC4691093

[8] Kirsch K, Heymel S, Gunther A et al (2021) Prognostication of neurologic outcome using gray-white-matter-ratio in comatose patients after cardiac arrest. BMC Neurol 21(1):456. 10.1186/s12883-021-02480-6 34809608 10.1186/s12883-021-02480-6 PMC8607613

[9] Koo TK, Li MY (2016) A guideline of selecting and reporting intraclass correlation coefficients for reliability research. J Chiropr Med 15(2):155–63. 10.1016/j.jcm.2016.02.012 27330520 10.1016/j.jcm.2016.02.012 PMC4913118

[10] Lancaster MA, Olson DV, McCrea MA, Nelson LD, LaRoche AA, Muftuler LT (2016) Acute white matter changes following sport-related concussion: a serial diffusion tensor and diffusion kurtosis tensor imaging study. Hum Brain Mapp 37(11):3821–3834. 10.1002/hbm.23278 27237455 10.1002/hbm.23278 PMC6867350

[11] Landis JR, Koch GG (Mar1977) The measurement of observer agreement for categorical data. Biometrics 33(1):159–174 843571

[12] Maas AIR, Menon DK, Adelson PD et al (2017) Traumatic brain injury: integrated approaches to improve prevention, clinical care, and research. Lancet Neurol 16(12):987–1048. 10.1016/S1474-4422(17)30371-X 29122524 10.1016/S1474-4422(17)30371-X

[13] Pinggera D, Rhomberg P, Beer R, Thome C, Petr O (2022) Brain tissue damage induced by multimodal neuromonitoring in situ during MRI after severe traumatic brain injury: incidence and clinical relevance. J Clin Med. 10.3390/jcm11113169 35683575 10.3390/jcm11113169 PMC9181231

[14] Pinggera D, Steiger R, Bauer M et al (2021) Repeated ^31^ P-Magnetic Resonance Spectroscopy in Severe Traumatic Brain Injury: Insights into Cerebral Energy Status and Altered Metabolism. Journal of neurotrauma 38(20):2822–2830. 10.1089/neu.2021.0143 34235953 10.1089/neu.2021.0143

[15] Richter S, Winzeck S, Kornaropoulos EN et al (2021) Neuroanatomical substrates and symptoms associated with magnetic resonance imaging of patients with mild traumatic brain injury. JAMA Netw Open 4(3):e210994. 10.1001/jamanetworkopen.2021.0994 33734414 10.1001/jamanetworkopen.2021.0994 PMC7974642

[16] Shakir A, Aksoy D, Mlynash M, Harris OA, Albers GW, Hirsch KG (2016) Prognostic value of quantitative diffusion-weighted MRI in patients with traumatic brain injury. J Neuroimaging 26(1):103–108. 10.1111/jon.12286 26296810 10.1111/jon.12286

[17] Stiefel MF, Spiotta A, Gracias VH et al (2005) Reduced mortality rate in patients with severe traumatic brain injury treated with brain tissue oxygen monitoring. Journal of neurosurgery 103(5):805–11. 10.3171/jns.2005.103.5.0805 16304983 10.3171/jns.2005.103.5.0805

[18] Wostrack M, Mielke D, Kato N et al (2015) Interobserver variability in the characterization of giant intracranial aneurysms with special emphasis on aneurysm diameter and shape. Acta Neurochir 157(11):1859–65. 10.1007/s00701-015-2587-1 26395008 10.1007/s00701-015-2587-1

